# Review on the Relationship between Human Polyomaviruses-Associated Tumors and Host Immune System

**DOI:** 10.1155/2012/542092

**Published:** 2012-03-25

**Authors:** Serena Delbue, Manola Comar, Pasquale Ferrante

**Affiliations:** ^1^Laboratory of Transkìlational Research, Health Science Foundation “Ettore Sansavini”, Corso Garibaldi, 11-48022 Lugo, Italy; ^2^Department of Reproductive, Developmental and Public Health Sciences, University of Trieste, Via dell'Istria 65/1, 34137 Trieste, Italy; ^3^Institute for Maternal and Child Health IRCCS “Burlo Garofolo”, Via dell'Istria 65/1, 34137 Trieste, Italy; ^4^Department of Public Health, Microbiology, Virology, University of Milano, Via Pascal 36, 20133 Milan, Italy; ^5^Istituto Clinico Città Studi, Via Ampere, 47, 20133 Milano, Italy

## Abstract

The polyomaviruses are small DNA viruses that can establish latency in the human host. The name polyomavirus is derived from the Greek roots *poly*-, which means “many,” and -*oma*, which means “tumours.” These viruses were originally isolated in mouse (mPyV) and in monkey (SV40). In 1971, the first human polyomaviruses BK and JC were isolated and subsequently demonstrated to be ubiquitous in the human population. To date, at least nine members of the *Polyomaviridae* family have been identified, some of them playing an etiological role in malignancies in immunosuppressed patients. Here, we describe the biology of human polyomaviruses, their nonmalignant and malignant potentials ability, and their relationship with the host immune response.

## 1. The Human Polyomaviruses

Polyomavirus is the sole genus of viruses within the *Polyomaviridae *family. Initially, polyomaviruses were taxonomically classified as a genus of the *Papovaviridae* family along with papillomaviruses; in 2000, however, the International Committee on Taxonomy of Viruses formally split the two viruses into two new families, *Polyomaviridae* and *Papillomaviridae* [1].

The name polyomavirus is derived from the Greek roots *poly-*, which means “many,” and -*oma*, which means “tumours.”

The first polyomavirus isolated was the mouse polyomavirus (mPyV) [2] causing the formation of multiple tumour types when inoculated into newborn mice. Subsequently, many polyomaviruses have been found to infect a variety of vertebrate species, including rabbits, rodents, birds, nonhuman primates, and, to date, nine polyomaviruses have been found in humans. The range of host species for each polyomavirus is very narrow, and the productive infection is limited to natural host [3].

Until 2006, BK virus (BKV) and JC virus (JCV), first isolated in 1971 [4, 5], were the only two human polyomaviruses known although some evidence suggested that the simian virus 40 (SV40) could be linked to some human tumours [6].

JCV and BKV usually infect the human population during early childhood, and primary infection is often asymptomatic. These viruses can remain latent in the kidney cells of the host until reactivation which occurs during immunodepression. JCV causes progressive multifocal leukoencephalopathy (PML) in cases of severe immunodeficiency, generally due to HIV infection, whereas BKV causes nephropathy in patients who have undergone to kidney transplants [7, 8]. SV40 does not infect naturally humans, but it was unintentionally introduced in the human population through contaminated polio vaccine in the late 1950s [6].

Lymphotropic polyomavirus (LPV) is another monkey polyomavirus that may also infect humans, since specific antibodies against it are present in the human population, and LPV DNA was detected in blood from immunosuppressed and immunocompetent subjects [9–11].

In 2007, two new human polyomaviruses were independently described: the KI polyomavirus (KIV) at Karolinska Institute and WU polyomavirus (WUV) the at the Washington University [12, 13]. These viruses that are closely related to each other were both identified from nasopharyngeal aspirates from children with respiratory tract infections. So far, no specific diseases have been associated to their infections. In 2008, a fifth polyomavirus, Merkel cell polyomavirus (MCPyV), was isolated from the skin of a patient affected by Merkel Cell carcinoma (MCC) showing its ability to cause most of the Merkel skin cancers [14]. In addition, three other polyomaviruses were isolated from no-tumoral skin, the Human Polyomavirus 6 and 7 (HPyV6, HPyV7), and the Trichodysplasia Spinulosa-associated Virus (TSPyV) [15, 16]. Finally, this year, the last polyomavirus, named Human Polyomavirus 9 (HPyV9), closely related to LPV, was identified from the blood and urine of asymptomatic renal transplant recipients [17] (Table 1).

**Figure 1:**
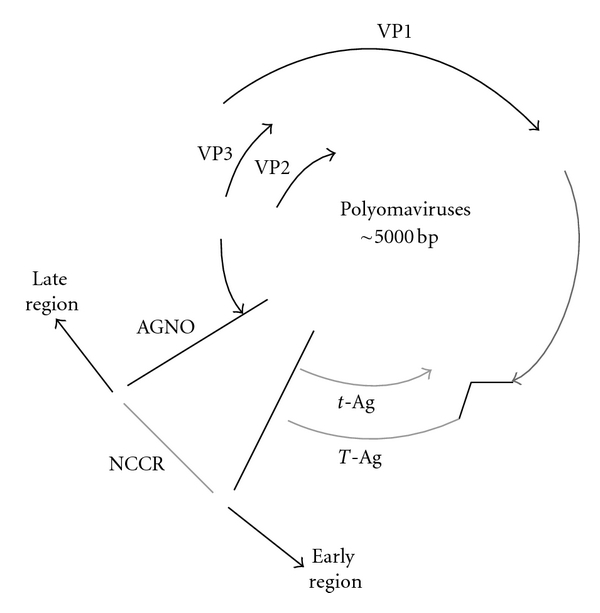
Schematic representation of the polyomaviruses genome organization. The circular double-stranded DNA genome is length about 5kb. The early region encodes the functional proteins LTAg and small T-Antigen, while the late region encodes the structural proteins VP1-3. The genome of JCV, BKV, and SV40 also encodes a small structural protein named Agno. The noncoding control region (NCCR) contains the origin of replication and regulates the replication and transcription of both the early and late genes.

Viruses that belong to the *Polyomaviridae* family are small and nonenveloped. They have icosahedral capsids, measuring 40.5–44 nm in diameter and circular, double-stranded supercoiled DNA genomes of approximately 5 Kb. The genome can be divided into two regions, one encoding the early proteins (the large and small Tumor Antigens) and another encoding the late, structural proteins Viral Proteins 1–3, which make up the capsid. The late region of BKV, JCV, and SV40 also encodes an agnoprotein, that seems to be implicated in viral maturation and microRNAs, which show a regulatory function in the expression of the Large Tumor Antigen (LTAg) [18, 19]. The early and late regions are separated by a noncoding region, the transcriptional control region (NCCR), which contains the origin of replication and promoters of viral transcription [20] (Figure 1). 

The primary infection of polyomaviruses is usually asymptomatic, probably occurring via respiratory and/or feco-oral transmission and establishing a latent phase of infection in the host. JCV and BKV persist in the kidney, while lymphoid tissue and the central nervous system (CNS) [21–23] have been indicated as possible sites of latency. HPyV6, HPyV7, TSPyV probably persist in the skin and in the lymphocytes, but little is known about the cell tropism of these most recent discovered viruses [24]. 

Under normal conditions polyomaviruses probably develop mechanisms to evade immune recognition and enable their latency into their human hosts, but these mechanisms are unknown. Very recently, Bauman and colleagues demonstrated that a viral miRNA identical in sequence between JCV and BKV is involved in immune regulation, resulting in the escape of the infected cells from the natural killer cells-mediated killing. In particular, it has been showed that the miRNA of polyomaviruses targets and downregulates the stress-induced ligand ULBP3, which is normally recognized by the killer receptor NKG2D, expressed by NK cells and other immune cells [25]. Consequently, the reduction of ULBP3 results in decreased killing of the infected cells [26]. 

Thus, reactivation of the infection is closely linked to the impairment of the immunological state of the host. There are two possible outcomes to infection of a cell by polyomaviruses. A productive infection occurs when polyomaviruses infect permissive cells: in this case, the virus entry into target cells is characterized by nuclear DNA replication and assembly of the viral capsid followed by the cell lysis. On the contrary, nonproductive infection is established when the virus infects nonpermissive cells blocking the viral DNA replication and triggering cell transformation (oncogenesis) [27]. 

## 2. Seroprevalence Polyomaviruses in the Human Population 

Polyomaviruses are widely distributed in the worldwide population, and their prevalence has been thoroughly investigated. First, it should be emphasized the high degree of identity between the genome and aminoacidic sequences of the human polyomaviruses VP1. For instance, the closely related viruses BKV and JCV share 78.2% identity of the VP1 amino acid sequences and even greater identity (81.3%) is shared between BKV and SV40. KIV and WUV show 66% of sequence homology and are phylogenetically compared with LPV. They are more distant to JCV and BKV presenting only 28% of amino acid identity [28–30]. Consequently, results of seroepidemiological studies to determine the prevalence of antibodies against specific human polyomaviruses could be highly heterogeneous considering their possibility to cross-react with all polyomaviruses species. In addition, the methods used to measure the specific seroreactivity to human polyomaviruses are different, including virus particles antigen assay, enzyme, linked immunosorbent assay (ELISA), hemoagglutination inhibition assay, and immune electron microscopy [31]. 

In general, serological population surveys for the detection of antibodies indicates that seroconversion to human polyomaviruses takes place early in life, 5 to 7 years for BKV, with conversion to JCV occurring later. Little is known about the occurrence of the exposure for the other newly discovered human polyomaviruses [30, 32–35]. Kean and colleagues determined the seroprevalence for LPV (14%), MCPyV isolate 350 (23–34%), KIV (56%), and WUV (54%) in a group of 721 young children, confirming that also, for these viruses, the seroconversion may occur during the childhood [9]. Serological evidence of exposure to MCPyV in childhood has been also reported by Chen et al. who observed a seroprevalence of 35% in children 4 to 13 years of age and by Tolstov et al. who described a seroprevalence of 34% in subjects under the age of 21 [36, 37]. The seroprevalence of TSPyV was estimated as 5% among children aged 1–4 years, rising to 48% at 6–10 years [38]. 

The reported adult levels of seroprevalence vary between 40 and 95% for BKV, between 32 and 95% for JCV, between 54% and 91% for both KIV and WUV, and between 25 and 88% for MCPyV [9, 35, 39–43]. LPV and LPV-related virus were reported to be present in the human population at lower level, about 15–20% of adults [9]. Only few seroprevalence data are reported for SV40 testing around 2–10% in the different geographic areas [44, 45]. 

## 3. Tumor Association of Human Polyomaviruses 

Research on the role of polyomaviruses in development of neoplasms began in 1953 when Gross isolated a virus, from a mice lymphoma, that induced an identical tumour when infected into newborn mice [46]. The virus, identified as murine polyomavirus (mPyV), became the archetypal member of *Polyomaviridae* family [2]. 

The ability of the polyomavirus SV40 to transform different cell types from many species and to cause tumours in animals model was described in 1960 [47]. To date, the transforming properties of SV40 have been well characterized and they have been used as models to study the potential oncogenicity of all other human polyomaviruses. 

Polyomaviruses have been found associated with specific tumor types such as brain and bone tumors, mesotheliomas, and lymphomas and with kidney diseases. Specifically, they can reproduce different types of brain tumours (e.g., medulloblastomas, neuroblastomas, astrocytomas, and neuroectodermal primitive tumours) when intracerebrally inoculated in rodents and monkeys [48, 49]. 

**Figure 2:**
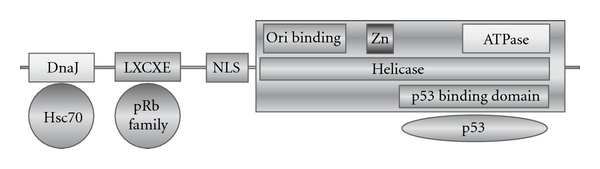
Polyomaviruses LT Ag structure. The functional domains are defined as follows: DnaJ domain that binds cellular Hsc70, LXCXE motif that binds the proteins belonging to the pRb family, NLS domain, that is the nuclear organization signal, Helicase domain, and p53 binding domain, that binds the p53 cellular suppressor protein.

It has been demonstrated by several in vitro studies that the main factor implicated in cell transformation and tumour development is the early protein LTAg. LTAg is a multifunctional protein, fundamental for the viral life cycle of polyomaviruses, because it regulates the viral genome replication and gene expression [50]. The early regulatory protein LTAg is functionally divided in several domains, defined, from the N-terminal to the C-terminal, as follows: the DNaJ domain, linking to the cellular factor HSc70; the LXCXE motif, that specifically binds and inactivates the Rb family members; the NLS domain, that is necessary for the nuclear localization of the protein; the Helicase domain; finally, the p53 binding domain [27, 51–53] (Figure 2). All these domains cooperate in binding to and inactivating cellular proteins that prevent transition into S phase. Consequently, the virus, that needs the S phase in order to replicate, inhibits itself replication. 

Transgenic mice with the polyomaviruses LTAg gene under the control of the early viral promoter have developed brain tumours, such as neuroblastomas and medulloblastomas [54–56]. The transforming activity of LTAg protein is due to the fact that it can bind and inactivate cellular tumour suppressor proteins, such as p53 and pRb [57, 58]. Besides these mechanisms of tumourigenicity, which have been analyzed in depth, it has also been shown that LTAg is able to deregulate the cell cycle also by interacting with beta-catenin, a component of the Wnt signal pathway, and enhancing the expression of c-myc [59]. LTAg can bind IRS-1, which is translocated in the cell nucleus, where it cooperates in the process of malignant transformation [60]. Finally, LTAg can bind HSc70, which affects the cell cycle. 

Polyomaviruses LTAg is also able to promote mutagenic events and chromosomal instability in B lymphocytes and morphological changes, such as multinuclei, aneuplodia, and polyplodia in transgenic mice [61, 62]. 

LTAg DNA belonging to JCV, BKV, and SV40 has been found in different types of human tumours, such as brain tumours (medulloblastomas, astrocytomas, ependymomas, oligodendrogliomas, and glioblastomas), bone tumours, colorectal carcinoma, urinary tract tumours, and lymphomas. Moreover, the LTAg, expressed protein has also been identified and localized in almost all the types of above-mentioned tumours [63–67], showing that the virus is able to express the oncogenic protein in the host and to cooperate in cell transformation. 

The transforming properties of the very recently discovered KIV and WUV have been tested only by a few groups, but the results are preliminary. On the contrary, MCPyV, since its discovery, has been seriously thought as a candidate etiological agent in the development of Merkel Cell Carcinoma (see below) (Table 2). 

## 4. Immunity to Polyomaviruses-Induced Tumours: The Polyomavirus Mouse Model 

The role of the immune surveillance in polyomaviruses reactivation is essential, since the virus-induced pathology mainly occurs in immunocompromised hosts. However, except for molecular-epidemiological studies, there is little information about the role of innate immunity related to polyomaviruses, and how the innate immune system handles the virus, whereas more information could be obtained regarding the adaptive immunity. 

In addition, because polyomaviruses have a narrow host range that restricts productive infection to their natural hosts, the only tractable system for studying polyomaviruses pathogenesis and immunity is the mPyV infection model. Since its discovery and given the ease of genome manipulation and expansion in tissue cultures, the mPyV has been used as infection model for the human polyomaviruses. When inoculated into immunocompromised adult mice or newborn mice of different inbred strains, polyomavirus induces multiple tumours that may arise from more than a dozen different cell types [68, 69]. 

The main study on the role of the innate immune response to the polyomaviruses infection in the contest of susceptibility to virus-induced tumours has been published several years ago, by Velupillai and colleagues [70, 71]. They studied two different inbred strains of mice, infected with mPyV. The first strain, known as BR mice, was resistant to the tumour, because of an antitumor immune response mediated by CD8^+^ T cells specific for a peptide derived from the viral middle T-antigen. The effective production of IL-12 and of IFN-gamma allowed an effective cell-mediated immunity. The second strain, indeed, called PE mice, was highly susceptible to the tumour and transmitted its susceptibility as a dominant trait in crosses with BR mice. This susceptibility was shown to be due to the absence of a type 1 cytokine response, leading to a failure to sustain virus-specific cytotoxic T-lymphocytes. PE mice, in fact, secreted IL-10, with no detectable production of IFN-gamma, and, only after administration of IFN-gamma and recombinant IL-12 to newborn mice, cytotoxic activity was restored and a reduction in tumours frequency was achieved [72]. 

In addition, Lowe and colleagues demonstrated the importance of the innate immunity in increasing the immune response against SV40 LTAg, using mice model, affected with SV40 induced-pulmonary metastasis. They reported upregulation of expression of inflammatory cytokines, such as TNFalpha, IL4, IL2, and RANTES, after tumorigenic growth and observed that it was correlated with beneficial reactions. Probably, these inflammatory mediators induced NK cells activation that led to the destruction of malignant cells [73]. 

The role of NK cells and *γδ*T cells in killing tumor cells was also investigated by Mishra in colleagues in 2010. They observed that mice lacking *αβ*T cells are protected from the formation of tumors induced by the mPyV if they have *γδ*T cells and NK cells. In addition, mice lacking both cells develop the tumors earlier than mice that have only NK cells. Additional experiments showed that NK cells and *γδ*T cells mount antitumor but not antiviral responses, since they do not have any effects on the amount of persisting virus [74]. 

Many studies have indicated that multiple components of the immune system contribute to limiting mPyV replication, including early induction of antibodies, recruitment of CD4^+^ and CD8^+^ T cells, and generation of humoral immunity. 

Firstly, it has been observed that congenitally thymus-deprived nude mice have increased susceptibility to virus oncogenesis, demonstrating that T cells prevent the tumours induction. Then, the results were enriched by the observation that knockout mice with complete lack of major histocompatibility complex (MHC) class I molecules were mPyV tumour-susceptible. Consequently, MHC-class I-restricted CD8^+^ T cells are thought to be the primary effectors-cell population, during polyomaviruses infection [75, 76]. 

Since polyomaviruses infection is persistent in the host, the immune system has to face with repetitive antigen encounter. In this situation of chronic antigen exposure, host mechanisms may come into play to intentionally downregulate CTL activity to prevent widespread destruction of antigen-bearing cells. Thus, peripheral tolerance mechanisms are engaged to deregulate antigen-specific CD8^+^ T cell responses. Many models of tolerance come from pathogenic persistent viruses in humans, including HCV, EBV, HIV, and HBV [77–80]. In these cases, CD8^+^ T cells gradually lack functional competence and weaken their cytotoxicity. However, the premature termination of antiviral CD8^+^ T cell function for oncogenic viruses can cause serious consequences. In transgenic mice, in fact, T-cell response is compromised by either central or peripheral tolerance [81, 82]. 

The role of CD4^+^ T lymphocytes in immunity to virus-induced tumours has been less studied, but it is thought to provide help for MHC-class I-restricted CD8^+^ cytotoxic (antitumour) T lymphocytes. Kennedy et al. evaluated the role of T-cell subsets in tumour immunity induced by recombinant SV40 T-Ag within an experimental murine pulmonary metastasis model. By depleting mice of either CD4^+^ or CD8^+^ T cells, indications were found that CD4^+^ T cells but not CD8^+^ T cells were critical in the production of antibodies to LTAg and in tumour immunity. Then, it was shown that IgG1 was the dominating IgG subclass indicating that Th2 type T-helper cells were involved [83]. CD4^+^ T cells are also required for recruitment of naive antiviral CD8^+^ T cells during persistent mPyV infection [84]. Two dominant mPyV-specific CD4^+^ T cells populations are present, one directed toward an epitope derived from the LTAg and the other from the VP1, that differed quantitatively and qualitatively during the course of infection. They are both stably maintained during persistent infection, but the VP1-specific CD4^+^ T-cell response is higher than that against LTAg. In addition, mPyV-specific CD4^+^ T cells, although not essential for expansion of CD8^+^ T cells during acute infection, are indispensable for de novo priming of antiviral naive CD8^+^ T cells in persistently infected hosts [85]. 

The potential cytotoxic activity of polyomavirus-specific CD4^+^ T cells has been studied in BKV-seropositive subjects, who also showed BKV-specific CD4^+^ and CD8^+^ cells. After expansion in culture, in fact, the majority of the BKV-specific CD4^+^ T cells was shown to express CD40, to secrete both IFN-gamma and TNF-alpha, to contain both granzyme A and granzyme B, and to degranulate/mobilize CD107 in response to antigen-specific stimulation [86]. 

Moreover, tumour induction was found to be age dependent and this age-related resistance to tumour induction was immunologically mediated as animal models that have been immunocompromised by X-irradiation become susceptible to tumour induction. 

## 5. Potential Clinical Application of Immunotherapy 

One of the strategies that has been used for adoptive immunotherapy of cancer identifies, isolates, and expands in vitro CD8^+^ T-cells responsive to human tumours antigens. However, few immunotherapeutic approaches that target the recruitment of tumor reactive CD8^+^ T cells have been effective against solid tumors. Surely, tumors of the CNS provide a unique challenge to immunotherapy due to more stringent regulation of lymphocyte circulation and the potential for the negative side effects induced by T-cell effector functions. In 2008, a mouse model of autochthonous brain cancer to assess adoptive immunotherapeutic approaches of polyomavirus-induced disease was examined. This model consisted of SV40 LTAg transgenic mice SV11 which develop spontaneous choroid plexus tumors due to expression of full-length LTAg and lack the endogenous CD8^+^ T cells that in nontransgenic mice respond to the three different LTAg epitopes, I, II/III, and IV. In the model, donor T cells against the immunodominant epitope IV are subject to the potential effects of peripheral tolerance and the immunosuppressive tumor environment following adoptive transfer into SV11 mice with both minimal disease and advanced stage tumors. The performed experiments demonstrated that LTAg-specific CD8^+^ T cells from immune donors accumulated at the tumor site and are associated with reduced tumor burden and extension of the lifespan. In addition, it has been shown that IFN-gamma component donor cells play a major role in the immune-mediated control of established autochthonous tumors in CNS [87, 88]. 

Many other studies are needed to confirm the feasibility of immunotherapy approaches to polyomavirus-induced tumors. 

## 6. A Special Focus on the Merkel Cell Polyomavirus 

Feng and colleagues have published the identification of Merkel Cell Polyomavirus (MCPyV) DNA in the in Merkel Cell Carcinoma samples (MCC) in 2008. The innovative method used consists in generating cDNAs library from target cell transcripts, in pyrosequencing the DNA and finally in screening all the sequencing-data in order to identify non human transcripts. The detected non human sequence was a fusion transcript between an unknown virus LTAg and a human receptor tyrosine phosphatase. Further investigation by the same group led to the sequence of the complete genome of this previously unknown polyomavirus, the MCPyV [14]. 

MCC is a neuroectodermal tumour arising from mechanoreceptor Merkel Cells that are present in the skin of the limbs and face and around hair follicles [89]. It is a rare and aggressive skin cancer, unusual before the age of 50, with an incidence rate of 0.44 cases per 100,000 subjects in the USA; however, its incidence is dramatically increasing, tripling from 1986 to 2001. The risk factors for the development of MCC comprise excessive UV light exposure, age >50 years old and immunosuppressive state of the host [89–92]. 

The paper by Feng and colleagues reported the detection of sequences in 8 of 10 (80%) MCC tumours, but only in 9 of 84 (10,7%) control tissues, including skin tissue. In addition, the ability of the MCPyV to integrate into the human genome was described: in six of eight positive tissues, the MCPyV genome was integrated within the host genome in a clonally pattern; metastatic cells also carried the same integration pattern. On the contrary, genome was present in the episomal form in all the other nontumour positive tissues. All the above-mentioned virus characteristics led the scientific community to state that MCPyV may be a candidate causative agent in the etiopathogenesis of MCC. 

Moreover, it was observed in a subsequent study that the LTAg amplified from 9 out of 9 MCC tissues had a mutation, resulting in a truncated protein, that lacked the helicase domain. On the contrary, the wild-type form of LTAg was detected in non-MCC tissues [14, 93–96]. 

Since the initial report, many other studies, have been published around the world, confirming the presence of this polyomavirus in MCC. In particular, they refer that the range of virus recovering from MCC fresh, frozen, formalin-fixed, or paraffin-embedded tumour samples varies between 24% and 100%, even if the data cannot be compared because of the different methodologies employed [97]. 

The prevalence of MCPyV in normal tissues or tumours other than MCC are more contrasting. The virus genome has been detected in skin samples with variably frequency ranging from 10% to 30%, whereas other studies reported prevalence rates of McCoy DNA up to 96%, in cutaneous swabs [98]. The virus was also searched in samples from neuroendocrine tumours from a variety of anatomic sites, colorectal cancer, prostate cancer, hematopoietic neoplasm, central nervous system tumours, mesothelioma, and many others, but none of the studies showed an association between virus and disease. In addition, the reported numbers of virus genome copies per MCC tumour cell are much higher (range 0.8–173/cell) [95, 99] than the highest value obtained for the other MCPyV genome-positive samples (0.01–0.0001 genome copies/cell). Finally, the antibody titres against MCPyV are much higher in MCC patients compared to healthy controls [100, 101] 

The proposed mechanism by which MCPyV induces MCC in virus-positive tumours is based on the activity of the truncated LTAg: it retains the region with oncogenic potential such as the DnaJ domain and the retinoblastoma binding pocket but lacks the domain supporting the viral DNA replication. This is important because the loss of replication activity upon integration in MCC demonstrates that is not simply a “passenger virus” that happens to grow well in MCC cells [93, 96, 102]. Moreover, the immunoprecipitation assays have shown that truncated LTAg is able to bind to Rb protein, a critical step into carcinogenesis event. The production of LTAg is necessary for the proliferation of MCPyV-positive MCC, since it has been demonstrated that the silencing of the virus gene by short-hairpin RNA causes growth arrest and/or cell death of the specific cell lines. Thus, it has been proposed that MCPyV plays an important role in the MCC pathogenesis by means of viral integration into the host and specific mutations in the LTAg region that retains the Rb-binding capacity, eliminating the possibility of viral replication. 

## 7. Immunobiology of MCPyV-Positive MCC 

As for the other human polyomaviruses, a strong link between MCPyV-positive MCC and the immune system has been suggested. Patients with immune system impairment, such as HIV-related immunodeficiency, drugs-associated immunosuppression, or forms of chronic leukaemia, are at up to 50-fold increased risk of developing MCC [89, 90]. Additionally, it has been shown that MCC tumours regress following improvement in immune function. 

As already reported above, the high and wide seroprevalence of MCPyV suggests that the exposure to the virus is a common event in the human population and that the capsid virus proteins are well recognized by the host immune system. However, MCPyV-positive MCC patients show a higher titer of antibodies against VP1, compared to the healthy population or to MCPyV-negative MCC patients, probably due to the exposure to an higher MCPyV viral load. In addition, the presence of a strong humoral immune response to MCPyV has been associated with a better prognosis of the tumours, perhaps showing that host immune system plays a critical role also in the remission of the infection [103]. 

Interestingly, Paulson and colleagues collected sera from 205 MCC cases and from 530 healthy controls and screened them for T-Ag reactivity, demonstrating that IgG antibody reactivity to the MCPyV LTAg and st-Ag oncoproteins was significatively associated with MCC. In addition, they observed rapid changes in LTAg titre corresponding to the different disease burden: titres were highest when patients were sickest, suggesting that antibodies to LTAg are not protective against disease progression. The authors hypothesized that the rapid turnover of IgG antibodies recognizing LTAg in MCC patient may be due to the instability within the B-cell population, caused by ineffective priming within the tumour microenvironment [104]. In contrast to capsid proteins that are highly visible to the humoral immune system, T-antigen oncoproteins are not present in viral particles, are only expressed after viral entry into host cells, are located in the nucleus [105], and are thus less likely to trigger an antibody response except in the setting of dying or diseased tissue (such as a tumour that persistently expresses the LTAg). 

Very recently, it was observed that MCPyV-specific T-helper cells secrete the IL13, IL10 cytokines, and IFN-gamma, that have a strong tumour-suppressing and antiviral functions [106]. 

The specific immune response mediated by T cells has also been documented in MCPyV-positive MCC patients. A study published recently has shown that intratumoral infiltration of CD8^+^ lymphocytes is a factor of improved prognosis of the disease. The same study showed also an overexpression of genes encoding cytotoxic granules such as granzymes, chemokine CCL19, and many lymphocyte-activation molecules in patients with improved survival [107]. 

## 8. Conclusions 

The recent identification of many components of the *Polyomaviridae* family that are able to infect humans represents a strong incentive for the scientific community to improve and increase the research on the oncogenic potentialities of these viruses. The ubiquity and persistent nature of polyomaviruses make them very challenging in order to define the mechanisms of their pathogenicity. In particular, since the association between the viral aetiology of the tumours and the state of the host immune system is well established, it is urgent to focus future studies on the nature of the relationship between the host immune system and polyomaviruses infection. 

## Figures and Tables

**Table 1:** Human polyomaviruses.

Nomenclature	Year identified	Prevalence in human population	Disease associations
BKV	1971	>90% of adults	Cystitis, polyomavirus-associated nephropathies, ureteral stenosis
JCV	1971	>70% of adults	Progressive multifocal leukoencephalopathy
KIV	2007	55–70% of adults	Not defined
WUV	2007	69–80% of adults	Not defined
MCPyV	2008	42–70% of adults	Merkel cell carcinoma
HPyV6	2010	Not defined	Not defined
HPyV7	2010	Not defined	Not defined
TSPyV	2010	Not defined	Transplant-associated trichodysplasia spinulosum
HPyV9	2011	Not defined	Not defined

BKV: BK virus; JCV: JC virus; KIV: Karolinska Institute polyomavirus; WUV: Washington University polyomavirus; MCPyV: Merkel cell polyomavirus; HPyV6: human polyomavirus 6; HPyV7: human polyomavirus 7; TSPyV: trichodysplasia spinulosum polyomavirus; HPyV9: human polyomavirus 9.

**Table 2:** Polyomaviruses-associated tumors.

Virus	Cancer	Viral product
JCV	brain tumors	DNA, RNA, protein
	Lymphoma (Hodgkin disease)	DNA, protein
	Leukemias	DNA
	Colorectal carcinoma	DNA, protein
	Gastric cancer	DNA
	Lung cancer	DNA, RNA, protein
	Esophageal carcinomas	DNA, protein
	Prostate cancer	DNA, protein
	Tongue carcinoma	DNA, protein

BKV	Brain tumors	DNA, RNA, protein
	Bone tumors	DNA, RNA
	Insulinomas	DNA
	Kaposi's sarcoma	DNA
	Urinary tract tumors/bladder tumors	DNA, RNA, protein
	Adrenal adenoma	DNA
	Genital tumors	DNA
	Renal carcinoma	DNA
	Prostate cancer	DNA, protein

SV40	Brain tumors	DNA, RNA, protein
	Mesotheliomas	DNA, RNA, protein
	Bone tumors	DNA
	Lymphomas	DNA
	Leukemias	DNA
	Urotheliomas	DNA
	Breast cancer	DNA, protein

MCPyV	Merkel cell carcinoma	DNA, protein

KIPyV	Not done	—

WUPyV	Not done	—
